# Nicotinamide Impairs Entry into and Exit from Meiosis I in Mouse Oocytes

**DOI:** 10.1371/journal.pone.0126194

**Published:** 2015-05-04

**Authors:** Angelique Riepsamen, Lindsay Wu, Laurin Lau, Dave Listijono, William Ledger, David Sinclair, Hayden Homer

**Affiliations:** 1 School of Women’s & Children’s Health, University of New South Wales, Sydney, New South Wales, Australia; 2 School of Medical Sciences, University of New South Wales, Sydney, New South Wales, Australia; 3 Genetics Department, Harvard Medical School, Boston, Massachusetts, United States of America; 4 Glenn Labs for the Biological Mechanisms of Aging, Harvard Medical School, Boston, Massachusetts, United States of America; Florida State University, UNITED STATES

## Abstract

Following exit from meiosis I, mammalian oocytes immediately enter meiosis II without an intervening interphase, accompanied by rapid reassembly of a bipolar spindle that maintains condensed chromosomes in a metaphase configuration (metaphase II arrest). Here we study the effect of nicotinamide (NAM), a non-competitive pan-sirtuin inhibitor, during meiotic maturation in mouse oocytes. Sirtuins are a family of seven NAD^+^-dependent deacetylases (Sirt1-7), which are involved in multiple cellular processes and are emerging as important regulators in oocytes and embryos. We found that NAM significantly delayed entry into meiosis I associated with delayed accumulation of the Cdk1 co-activator, cyclin B1. GVBD was also inhibited by the Sirt2-specific inhibitor, AGK2, and in a very similar pattern to NAM, supporting the notion that as in somatic cells, NAM inhibits sirtuins in oocytes. NAM did not affect subsequent spindle assembly, chromosome alignment or the timing of first polar body extrusion (PBE). Unexpectedly, however, in the majority of oocytes with a polar body, chromatin was decondensed and a nuclear structure was present. An identical phenotype was observed when flavopiridol was used to induce Cdk1 inactivation during late meiosis I prior to PBE, but not if Cdk1 was inactivated after PBE when metaphase II arrest was already established, altogether indicating that NAM impaired establishment rather than maintenance of metaphase II arrest. During meiosis I exit in NAM-treated medium, we found that cyclin B1 levels were lower and inhibitory Cdk1 phosphorylation was increased compared with controls. Although activation of the anaphase-promoting complex-Cdc20 (APC-Cdc20) occurred on-time in NAM-treated oocytes, Cdc20 levels were higher in very late meiosis I, pointing to exaggerated APC-Cdc20-mediated proteolysis as a reason for lower cyclin B1 levels. Collectively, therefore, our data indicate that by disrupting Cdk1 regulation, NAM impairs entry into meiosis I and the establishment of metaphase II arrest.

## Introduction

Mammalian oocytes undergo a protracted and discontinuous developmental programme that begins during fetal life and is not completed until postnatal adulthood [1]. The majority of this time is spent in a prophase I-arrested state with an intact nucleus, termed the germinal vesicle (GV) in oocytes, equivalent to a late G2-phase arrest [2, 3]. Following an extended growth phase, GV-stage oocytes acquire the competence to resume and complete the first meiotic division (meiosis I) marked by GV breakdown (GVBD) and first polar body extrusion (PBE), respectively. Unlike somatic cells which enter interphase following exit from mitosis, it is crucial that oocytes side-step interphase following PBE, and immediately enter M-phase of the second meiotic division (meiosis II) thereafter becoming arrested for a second time at metaphase II [4, 5]. The metaphase II-arrested oocyte, or egg, is the fertilization-competent gamete that is released at ovulation and is indispensable for reproduction.

Sirtuins are a family of conserved NAD^+^-dependent substrate-specific protein deacetylases that impact multiple somatic cell pathways involved in cellular and organismal aging and metabolism [6]. Mammals express seven sirtuins, Sirt1-7, which possess a highly conserved NAD^+^ catalytic domain [6]. Although categorised as class III histone deacetylases (HDACs), sirtuins possess a host of additional non-histone targets. Furthermore, individual sirtuins have differing substrate profiles and sub-cellular localization patterns [6]. Unlike other HDACs, sirtuin-mediated deacetylation involves a unique enzymatic reaction requiring NAD^+^ cleavage into nicotinamide (NAM) and an ADP-ribose peptide-imidate intermediate, the resolution of which culminates in release of the deacetylated substrate [6]. Significantly, NAM acts a non-competitive pan-sirtuin inhibitor by reacting with the ADP-ribose peptide-imidate intermediate to reform NAD^+^ and the acetylated protein [6, 7]. Sirtuin activity can therefore be positively modulated by increasing NAD^+^ availability and negatively regulated through increasing NAM.

A number of studies have examined the effects of NAM on oocytes and embryos. In ascidian oocytes, which are blocked in metaphase of meiosis I, NAM prevented fertilization-induced completion of meiosis I by blocking inactivation of maturation-promoting factor, otherwise known as cyclin-dependent kinase 1 (Cdk1) [8]. Recently, NAM was found to have beneficial effects during *in vitro* aging of ovulated metaphase II-arrested mouse oocytes associated with reduction in both spindle elongation and cellular fragmentation [9]. Regarding effects on embryo development, earlier data found that NAM inhibited mouse blastocyst formation *in vitro* and subsequent post-implantation development [10]. More recent data have replicated these findings and importantly, also showed that inhibition of embryo development observed with NAM was closely mirrored by two Sirt1 inhibitors, Sirtinol and BML-210 [11], suggesting that NAM inhibits sirtuins in reproductive cells as in somatic cells. Further in support of this, NAM also abolished the ability of the highly potent Sirt1 activator, SRT1720, to protect the primordial follicle pool from the detrimental effects of diet-induced obesity in mice [12]. Here we investigated the effects of NAM during meiosis I of mouse oocytes. We find that NAM negatively impacts two key transitions, GVBD and the meiosis I-to-meiosis II transition but not the intervening period during meiosis I.

## Materials and Methods

### Ethics Statement

This research was carried out in accordance with the Australian Code of Practice for the Care and Use of Animals for Scientific Purposes and was approved by the Animal Care and Ethics Committee of the University of NSW (approval number AE14/42B).

### Oocyte Collection, Culture and Chemical Treatment

GV-stage oocytes were collected from the ovaries of hormonally primed 7–9 week old Swiss mice. Intraperitoneal injections of 7.5 IU pregnant mare serum gonadotropin (PMSG; Intervet) were given 44–46 h prior to euthanasia and ovarian dissection. Ovarian follicles were punctured to release fully-grown oocytes into minimum essential medium alpha (αMEM; Gibco) containing 50 μg/ml gentamycin sulphate (Sigma) and HEPES. To maintain meiotic arrest at the GV stage, medium was supplemented with 50 μM 3-isobutyl-1-methyl-xanthine (IBMX; Sigma) [13–15]. Cumulus cells were removed by pipetting, and denuded oocytes were randomly allocated to either control or treatment groups. In order to induce resumption of meiotic maturation, oocytes were washed out of IBMX by transferring them through sequential IBMX-free micro-drops of pre-warmed medium under mineral oil. For longer-term culture, oocytes were transferred to micro-drops of HEPES-free αMEM medium under mineral oil at 37°C in a humidified atmosphere of 5% CO_2_ in air.

For NAM treatment, oocytes were cultured in medium containing a final concentration of 10 mM NAM (Sigma) prepared from 2 M stock solutions in water for 4 hours prior to being washed out from IBMX-treated medium. Final concentrations for small molecule inhibitors were as follows: AGK2 (20 μM; Sigma), Ex527 (10 μM; Sigma) Flavopiridol (5 μM; Sigma).

### Immunoblotting

For sample collection, oocytes were washed in Milli-Q water, lysed in LDS sample buffer (Invitrogen), snap-frozen and stored at ­80°C. For blotting, samples were thawed on ice before adding reducing agent (Invitrogen) and heated at 95°C for 10 minutes. Proteins were resolved on pre-cast 4–12% Bis-Tris gels (Invitrogen) for 55 min at 200 V according to the manufacturer’s recommendations. Proteins were then transferred to PVDF membranes (Millipore) using the XL II Blot Module (Invitrogen). Following transfer, membranes were blocked for 1 h at room temperature (RT) in 3% BSA in TBS (25 mM Tris, 150 mM NaCl, pH 8.0) containing 0.05% Tween-20 (TBST). Membranes were then incubated overnight at 4°C with primary antibody in blocking solution followed by the appropriate HRP-conjugated goat secondary antibody (Bio-Rad) as the second layer. Antibodies against securin, cyclin B1 and Cdc20 were used as described previously [13–15]. Phosphorylated Cdk1 (Y15) was detected using a rabbit antibody (Cell Signalling Technology) as before [16]. HRP-conjugated secondary antibodies were detected using the ECL Prime chemiluminescence detection system (GE Healthcare). Protein bands were quantified using the ImageQuant LAS 4000 (GE Healthcare) and data normalised against maximal band intensity values as before [14, 15]. Actin served as an internal control to ensure even sample loading and gel transfer.

### Immunofluorescence and Confocal Microscopy

As described previously [13–15], oocytes were very briefly washed through PHEM solution (60 mM PIPES at pH 6.9, 25 mM HEPES, 10 mM EGTA, 2 mM MgCl_2_.7H_2_0), before being pre-permeabilised in 0.25% Triton X-100 (Sigma) in PHEM for 5–10 seconds at RT. Oocytes were then fixed in 3.7% paraformaldehyde (Sigma) in PHEM for 30 minutes before being permeabilised for 10 minutes in 0.25% Triton X-100 in PBS. After washing in PBS containing 0.5% BSA for 5 minutes at RT, non-specific binding sites were blocked by overnight incubation in PBS containing 3% BSA and 0.05% Tween-20 (blocking solution) at 4°C. The following morning, after being allowed to return to RT, oocytes were probed with primary antibodies.

For immunolabelling, human anti-centromere antibodies (ACA; ImmunoVision); mouse anti-*β*-tubulin (Sigma), and; rabbit anti-Mad2 (Covance) were used as the first layer. For the second layer, the following secondary antibodies were used: Alexa Fluor 546-labelled goat anti-human (Invitrogen) for detecting ACA; Alexa Fluor 633- or Alexa Fluor 488-labelled goat anti mouse (Invitrogen) for detecting *β* -tubulin and Alexa Fluor 488-labelled goat anti rabbit (Invitrogen) for detecting Mad2. DNA was labelled using Hoechst 33342 (1 μg/ml; bisBenzimide; Sigma) in PBS for 30–60 seconds at RT. Oocytes were then transferred to 1–2 μl micro-drops of PBS under mineral oil in glass bottom dishes for confocal imaging.

Images were captured using an A1 MP+ multiphoton confocal microscope (Nikon, Japan) equipped with a C-Apochromat 63×/1.2 NA water immersion objective, processed using NIS-Elements C acquisition and analysis software (Nikon, Japan), and assembled into panels using Adobe Photoshop.

### Statistical Analysis

At least three replicates of all experiments were performed. Statistical comparisons were made with Student’s *t*-test and Fisher’s exact test as appropriate using GraphPad Prism 5.0 software. Values of *P* < 0.05 were considered to be significant.

## Results

### Nicotinamide (NAM) Delays GVBD

Following release from the inhibitory follicular environment, fully-grown GV-stage oocytes denuded of surrounding cumulus cells spontaneously enter M-phase of meiosis I [2, 4, 17]. Entry into meiosis I is marked by GV breakdown (GVBD), a readily identifiable morphological change that can be robustly inhibited using phosphodiesterase inhibitors such as 3-isobuyl 1-methylxanthine (IBMX)[2, 13–15].

**Fig 1:**
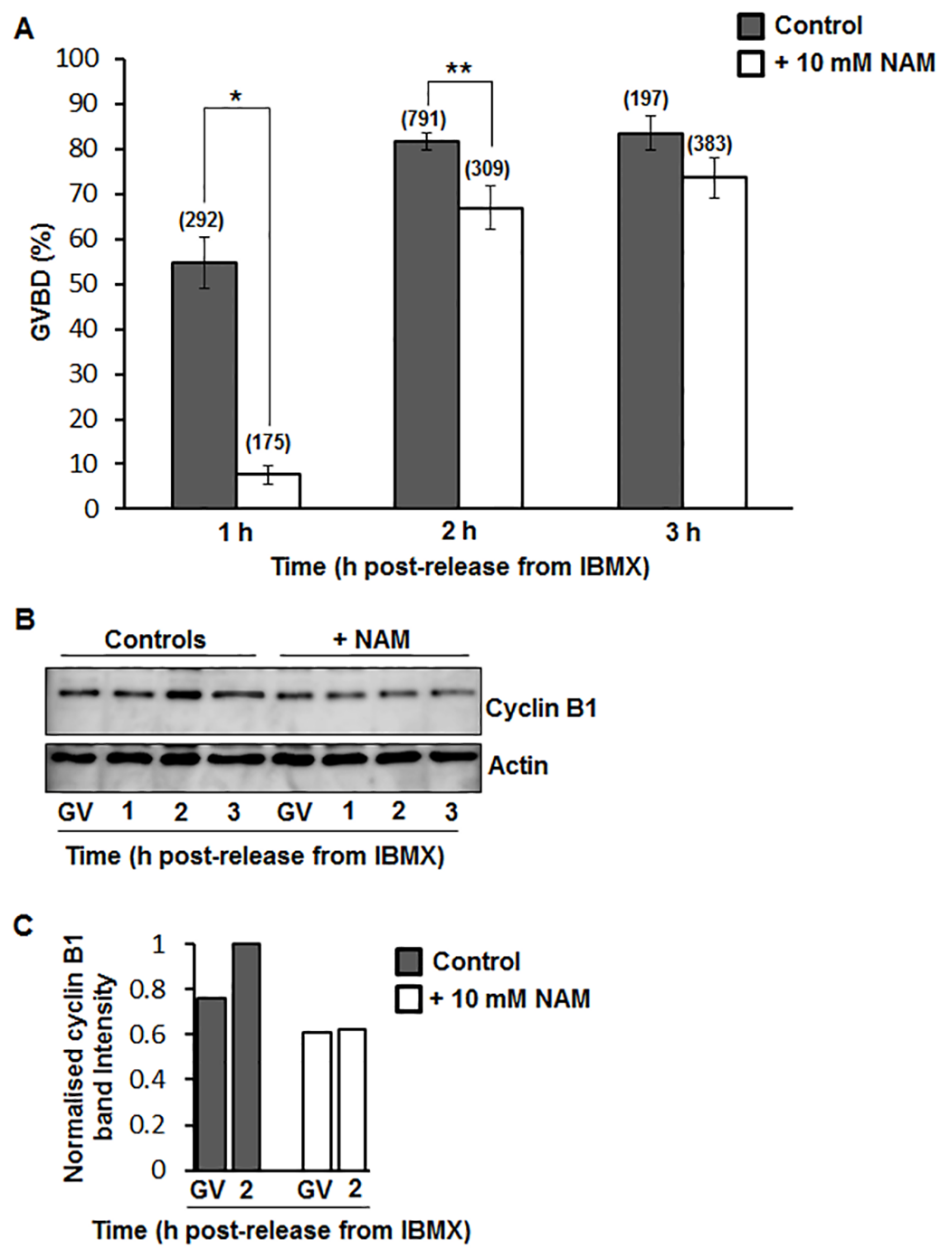
GVBD rates and cyclin B1 expression following release from IBMX. (A) Rates of GVBD were scored at 1, 2 and 3 h following release from IBMX in control untreated and NAM-treated oocytes (* *P* < 0.0001, ** *P* = 0.0011; Data are mean ± SEM; Analysis by Student’s *t*-test). Numbers of oocytes for each group are shown in parentheses and are pooled data from over 5 replicate experiments. (B) Western blot of cyclin B1 and actin at the GV-stage and at 1 h, 2 h and 3 h following release from IBMX (30 oocytes per sample; shown is a representative blot of 3 replicates). (C) Band intensities from Westerns were quantified and normalised against the intensity at 2 h post-release in control oocytes.

Fully-grown GV-stage oocytes were cultured in IBMX-supplemented media with or without 10 mM NAM. By 1 h post-release from IBMX, ~55% of controls underwent GVBD whereas GVBD rates were <10% in the NAM-treated group (Fig 1A). By 2 h post-release, control GVBD rates increased to ~80% and although by this stage GVBD had increased markedly in NAM-treated oocytes to 67%, this remained significantly lower than controls (Fig 1A). By 3 h after release from IBMX, NAM-treated oocytes continued to exhibit a trend towards reduced GVBD rates but this was no longer statistically significant (Fig 1A; 84% in controls versus 74% in NAM-treated; *P* = 0.14).

The concentration of NAM used here (10 mM) is one-fifth that found to inhibit meiotic completion in ascidian oocytes [8] and half the NAM concentration (20 mM) that was found to have a beneficial effect on ovulated mouse eggs [9]. Consistent with a dose-dependent effect for NAM, we found that 20 mM completely prevented GVBD at 1 h post-release from IBMX (0%; n = 54), and continued to reduce GVBD rates even after 3 h (33%; n = 99), when GVBD rates with 10 mM NAM were comparable to those of untreated controls (Fig 1A). Due to the less severe disruption caused by 10 mM NAM, we used this concentration for all subsequent experiments.

GVBD is underpinned by activation of the master kinase, Cdk1, brought about by increased levels of the Cdk1 activating subunit, cyclin B1 [2, 3]. We therefore examined changes in cyclin B1 around the time of GVBD using Western blotting. We found that in controls, cyclin B1 levels increased by over 20% between the GV-stage and 2 h post-release from IBMX when GVBD rates were near-maximal (Fig 1B and 1C). In contrast, in NAM-treated oocytes, cyclin B1 levels were lower than controls at the GV-stage and demonstrated very little increase over the ensuing 2 hours (Fig 1B and 1C). Thus, delayed GVBD following NAM treatment correlated with impaired cyclin B1 accumulation.

### Inhibition of Sirt2, but Not Sirt1, Delays GVBD in a Very Similar Pattern to NAM

**Fig 2:**
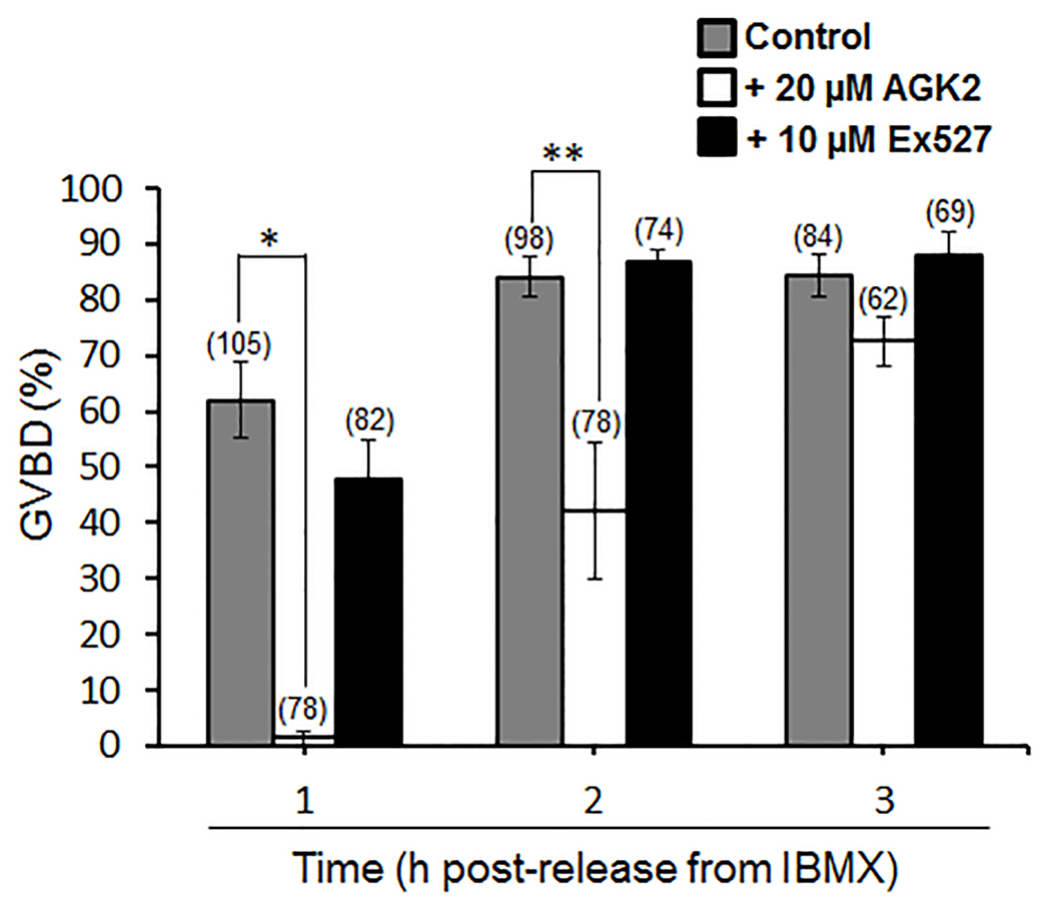
GVBD rates for AGK2- and Ex527-treated oocytes following release from IBMX. Fully-grown GV-stage oocytes were cultured in IBMX-containing medium treated with either 20 μM AGK2 or 10 μM Ex527 or left untreated. Rates of GVBD were scored at 1 h, 2 h and 3 h following release from IBMX (* *P* < 0.0001, ** *P* = 0.0002; Data are mean ± SEM; Analysis by Student’s *t*-test). Numbers of oocytes for each group are shown in parentheses and are pooled data from 3 replicate experiments.

Next we sought to determine whether inhibition of GVBD observed with NAM treatment reflected disruption of one (or more) sirtuin family members. Mammals express seven sirtuins, all of which are present in mouse eggs [11], so it was not feasible to pursue a gene knockdown approach for deciphering which sirtuin(s) might be mediating effects observed with NAM. Instead, we made use of two highly specific sirtuin inhibitors, AGK2 for inhibiting Sirt2 and Ex527 to inhibit Sirt1 [6, 18–20]. We used AGK2 and Ex527 at concentrations of 20 μM and 10 μM, respectively, in line with concentrations used previously in somatic cells [19] and mouse oocytes [18]. We found that AGK2 significantly delayed GVBD at 1 h and 2 h following release from IBMX but by 3 h, rates became comparable with control oocytes (Fig 2), very similar to the effects observed with NAM (Fig 1A). In contrast, Ex527 did not impact GVBD rates (Fig 2). Thus, inhibition of Sirt2, but not Sirt1, delayed GVBD in a very similar pattern to NAM. Given that in somatic cells, NAM is well known to inhibit sirtuins [6, 7], including Sirt2 [21], these data suggest that at least some of NAM’s effects on GVBD in oocytes reflect Sirt2 inhibition.

### Delayed Entry into Meiosis I Does Not Impact Spindle Assembly and Chromosome Alignment

**Fig 3:**
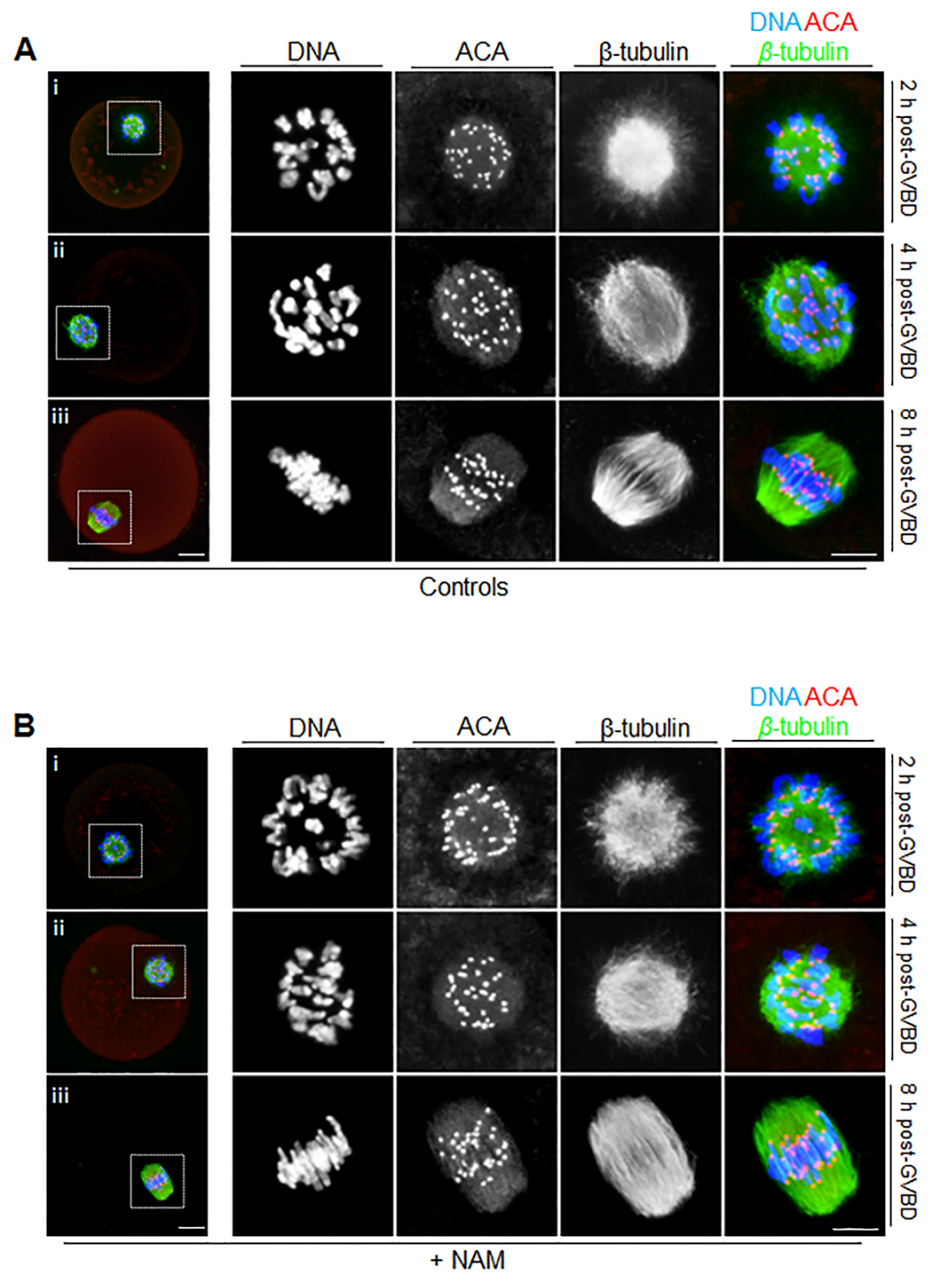
Spindle assembly and chromosome alignment during meiosis I. Immunofluorescence staining of oocytes for chromosomes (DNA), kinetochores (labelled using anti-centromere antibody; ACA) and spindle microtubules (β-tubulin) at 2 h, 4 h and 8 h post-GVBD, representing ball, intermediate and bipolar stages, respectively, in controls (A), and NAM-treated (B) oocytes. Panels to the extreme left are whole-oocyte images whilst panels to the right are magnified images of the regions enclosed by the dashed white squares. Scale bars = 10 μm.

Next we examined the effect of NAM on spindle assembly following GVBD by immunostaining oocytes at defined stages during meiosis I. In mouse oocytes, bipolar spindle assembly occurs in the absence of centrosomes and is a protracted process lasting 6–8 h [4, 22–24]. By 1–2 h post-GVBD, clumped chromosomes localize to the surface of a microtubule “ball”, the earliest spindle morphology during which kinetochore pairs of recombined homologous chromosomes lie adjacent to one another (Fig 3Ai). By 8 h post-GVBD, a barrel-shaped bipolar spindle has formed with chromosomes aligned at the spindle equator (Fig 3Aiii). At this stage, chromosomes have become stretched with kinetochore pairs directed towards opposite spindle poles. By 4 h post-GVBD, spindle assembly is intermediate between the above two extremes with chromosomes scattered throughout the spindle, some of which have become stretched (Fig 3Aii).

We found that NAM-treated oocytes consistently (12 of 12) established the earliest stage of spindle assembly, the ball spindle morphology, by 2 h post-GVBD (Fig 3Bi). Furthermore, none of the subsequent stages of spindle assembly were impacted by NAM treatment and oocytes attained intermediate- and bipolar-stages at 4 h and 8 h post-GVBD, respectively (Fig 3Bii and 3Biii) indistinguishable from controls (Fig 3Aii and 3Aiii). Overall therefore, NAM did not induce overt defects in spindle assembly and chromosome alignment during meiosis I.

### NAM-Treated Oocytes Undergo PBE on Schedule but Decondense Chromosomes and Form a Nuclear Structure

**Fig 4:**
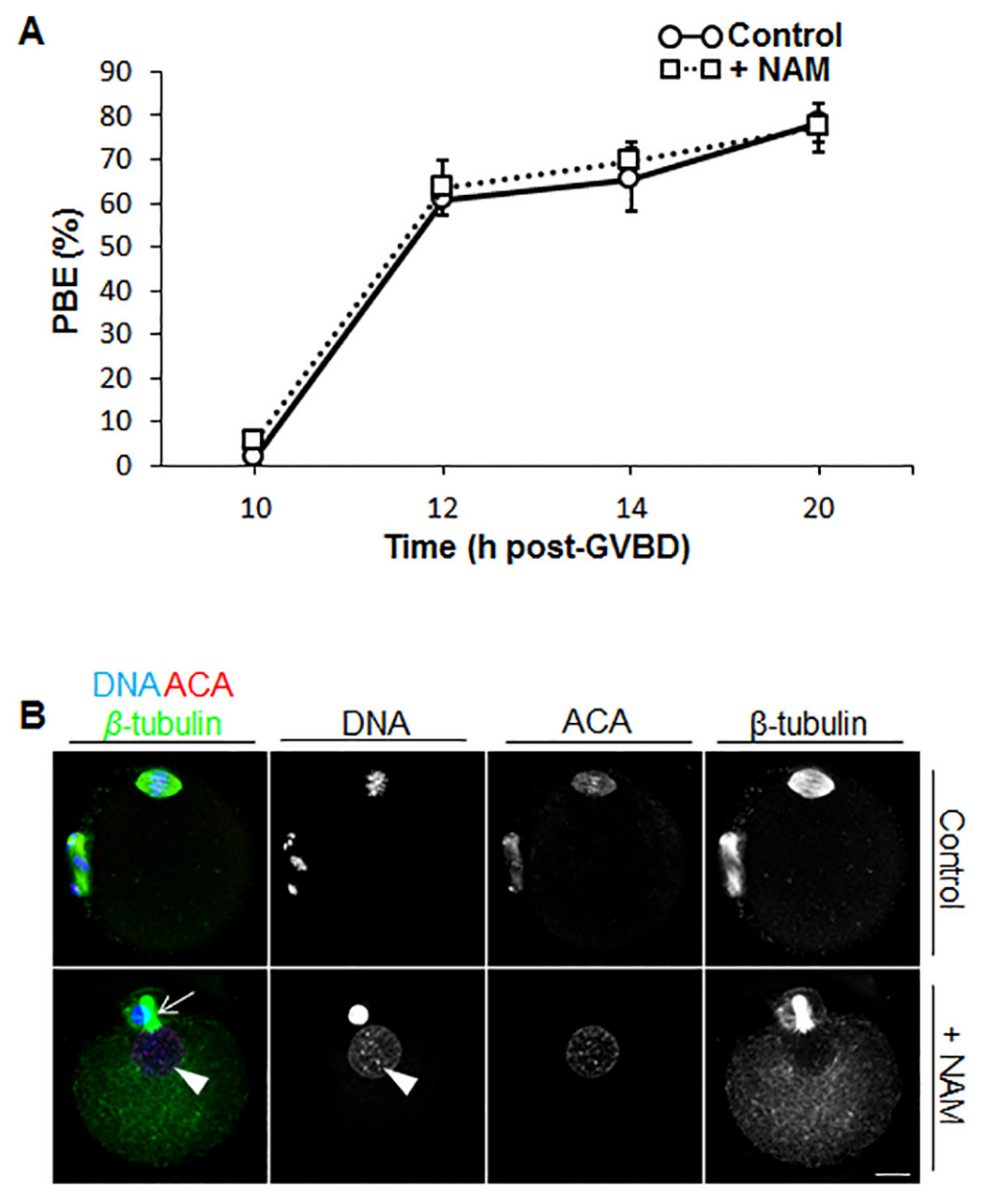
Polar body exclusion (PBE) rates and chromosome and spindle morphology in control and NAM-treated oocytes. (A) PBE rates were scored for control and NAM-treated oocytes at 10 h (n = 103 control and n = 90 NAM), 12 h (n = 104 control and n = 64 NAM), 14 h (n = 122 control and n = 60 NAM) and 20 h (n = 169 control and n = 131 NAM) post-GVBD. Numbers of oocytes are pooled data from 3 or more replicate experiments. Data are mean ± SEM. (B) Control and NAM-treated oocytes that exhibited a clear polar body were immunostained for chromosomes (DNA), kinetochores (ACA) and spindle microtubules (β-tubulin). Note the presence of the central spindle (white arrow) and decondensed chromatin enclosed within a nuclear structure (white arrow head) following NAM treatment. Scale bars = 10 μm.

Exit from meiosis I is marked by first polar body extrusion (PBE) after which oocytes immediately enter meiosis II and become arrested at metaphase II [4, 5]. About 60% of control oocytes underwent PBE by 12 h post-GVBD, rising to 65% and 78% by 14 h and 20 h post-GVBD, respectively (Fig 4A). Unlike the differences observed in GVBD with NAM treatment, PBE rates were indistinguishable from controls, being 63%, 70% and 77% at 12 h, 14 h and 20 h post-GVBD, respectively (Fig 4A).

Unexpectedly, although PBE rates were unperturbed, immunostaining revealed striking defects in spindle and chromosomal organisation following NAM treatment. Control oocytes with clearly visible PBs exhibited the typical bipolar spindle with condensed chromosomes that were tightly aligned at the metaphase II plate (n = 21; Fig 4B). In marked contrast, we found that the majority (~71%; n = 31) of NAM-treated oocytes with clear PBs lacked a metaphase II-arrested phenotype. In 32% of these cases, spindles were improperly formed and chromosomal arrangement was disorganised. Strikingly, in the most frequent abnormality, oocytes exhibited nuclei containing decondensed chromatin and completely lacked a bipolar spindle, instead forming a network of microtubules that radiated throughout the ooplasm (Fig 4B), a phenotype we never observed in control oocytes (*P* < 0.0001). A prominent feature in such oocytes was the persistence of the central spindle, which in conjunction with DNA present in both the oocyte and the PB (Fig 4B), indicated that anaphase I had occurred, but that subsequent events were severely impaired. Taken together therefore, these findings indicate that NAM treatment impairs late meiosis I events after anaphase I.

### Impairment of the Meiosis I-To-Meiosis II Transition Is Not Associated with Impaired APC/C Activation in Late Meiosis I

The phenotype observed with NAM treatment comprised of a single PB, a persisting central spindle and a nuclear structure containing decondensed chromatin (Fig 4B) was reminiscent of that observed very recently in oocytes lacking microtubule-associated serine/threonine kinase like (Mastl) [16]. *Mastl* ^*-/-*^ oocytes exhibit impaired activation of the Cdc20-activated species anaphase-promoting complex (APC-Cdc20), which leads to delayed destruction of key APC-Cdc20 substrates required for exit from meiosis I [16].

**Fig 5:**
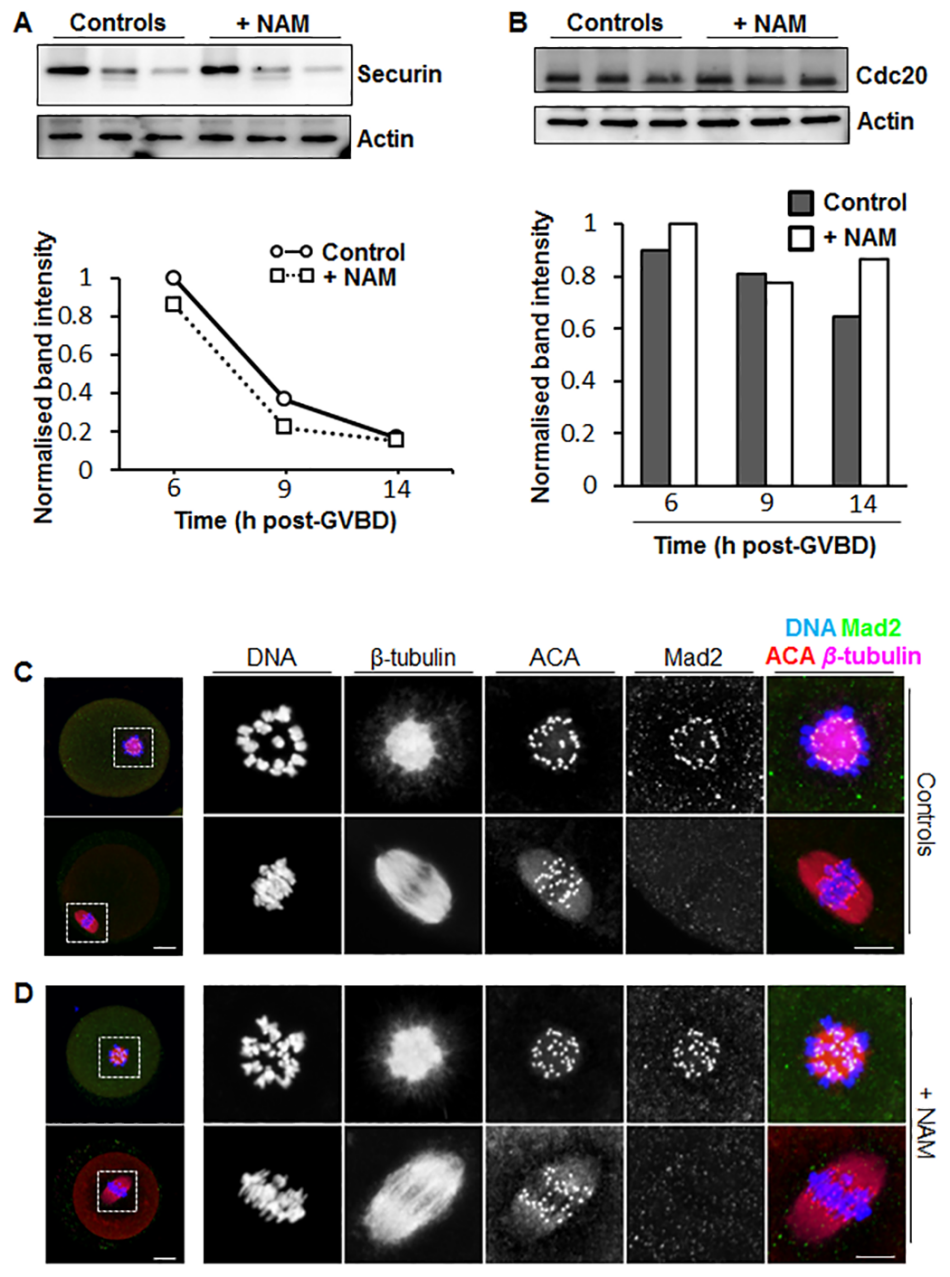
Effect of NAM treatment on securin and Cdc20 levels and kinetochore Mad2 localisation during meiosis I. (A) Western blot of securin and actin in control and NAM-treated oocytes at 6 h, 9 h and 14 h post-GVBD (30 oocytes per sample; shown is a representative blot of 3 replicates). Band intensities from Westerns were quantified and normalised against the maximal intensity at 6 h post-GVBD and plotted as a graph. (B) Western blot of Cdc20 and actin in control and NAM-treated oocytes at 6 h, 9 h and 14 h post-GVBD (30 oocytes per sample; shown is a representative blot of 3 replicates). Band intensities from Westerns were quantified and normalised against the maximal intensity at 6 h post-GVBD. (C, D) Changes in kinetochore Mad2 levels during meiosis I. Immunofluorescence staining for chromosomes (DNA), kinetochores (ACA), Mad2 and spindle microtubules (β-tubulin) at 2 h (upper panels) and 8 h (lower panels) post-GVBD in controls (C), and NAM-treated (D) oocytes. Note that at 2 h post-GVBD when spindles are at the ball stage, kinetochores recruit high levels of microtubules and that by 8 h post-GVBD when a bipolar spindle is present, Mad2 is undetectable at kinetochores in both groups. Panels to the extreme left are whole-oocyte images whilst panels to the right are magnified images of the regions enclosed by the dashed white squares. Scale bars = 10 μm.

This prompted us to examine APC-Cdc20 activation during late meiosis I in NAM-treated oocytes. In mouse oocytes, destruction of two APC-Cdc20 substrates, securin and cyclin B1, are required for exit from meiosis I [25, 26]. The pattern of securin destruction has therefore been used as a surrogate marker of APC-Cdc20 activity in mouse oocytes with the onset of securin destruction correlating with initial APC-Cdc20 activation [16, 27]. Using immunoblotting we found that in control oocytes, securin declined precipitously between 6 h and 9 h post-GVBD, with a further decline by 14 h post-GVBD (Fig 5A), indicating that APC-Cdc20-mediated destruction commences between 6 h and 9 h post-GVBD. Notably, securin levels also declined markedly from 6 h to 9 h post-GVBD, with an additional decline from 9 h to 14 h post-GVBD, in NAM-treated oocytes (Fig 5A). Moreover, the pattern of decline in NAM-treated oocytes was similar to that in controls (Fig 5A).

APC-Cdc20 activity in oocytes is determined by the levels of its Cdc20 co-activator [26, 28]. We therefore examined Cdc20 levels at 6 h, 9 h and 14 h post-GVBD, spanning the period of protein destruction we observed. If NAM treatment impaired APC-Cdc20 activity we might expect lower Cdc20 levels in NAM-treated oocytes. This was not the case however, as Western blotting showed that Cdc20 levels were not consistently lower in NAM-treated oocytes (Fig 5B). Indeed, there was a trend towards increased levels of Cdc20 at two of the three time-points following NAM treatment (Fig 5B), which might account for lower securin levels during exit from meiosis I (Fig 5A). Overall therefore, securin destruction profiles and Cdc20 levels were not indicative of either delayed APC-Cdc20 activation or reduced APC-Cdc20 activity in NAM-treated oocytes.

The timing of APC-Cdc20 activation is controlled by the spindle assembly checkpoint (SAC) surveillance mechanism. As another means for investigating APC-Cdc20 activation following NAM treatment, we therefore examined the SAC. Kinetochores are the platform upon which the inhibitory SAC signal is generated [29, 30]. Mad2 is a critically important SAC protein whose activation at unattached kinetochores promotes its ability to bind Cdc20 and so prevent APC activation [30]. Prior to the formation of attachments between spindle microtubules and kinetochores during early meiosis I, we found that kinetochores in both control and NAM-treated oocytes recruited high levels of Mad2 (Fig 5C and 5D, upper panels). By late meiosis I, after stable attachments have become established [13, 31], kinetochore Mad2 levels declined precipitously in both groups of oocytes (Fig 5C and 5D, lower panels). Thus, Mad2 displacement from kinetochores, and hence SAC inactivation, is not perturbed in NAM-treated oocytes.

In conclusion, on the basis of the timing of securin destruction, Cdc20 levels and SAC inactivation, severe defects at the meiosis I-to-meiosis II transition following NAM treatment were not associated with defective or reduced APC-Cdc20 activation unlike the case with *Mastl* ^*-/-*^ oocytes[16].

### The Defect at the Meiosis I-To-Meiosis II Transition following NAM Treatment Reflects a Failure to Establish, Rather than to Maintain, a Metaphase II Arrest State

The metaphase II-arrested state is characterised by sustained Cdk1 activity, which maintains chromosomes in a condensed state, promotes bipolar spindle assembly and prevents nuclear envelope reassembly [5]. The phenotype induced by NAM treatment characterised by decondensed chromatin, a nuclear structure and a diffuse network of microtubules therefore suggested that oocytes were in an interphase-like state, pointing to marked Cdk1 inactivation.

Two scenarios could potentially account for the NAM phenotype, either oocytes failed to establish a metaphase II arrest and exited meiosis I into an interphase-like state or oocytes initially established a metaphase II arrest but then failed to maintain it as occurs with parthenogenetic activation. For instance, oocytes lacking either Early mitotic inhibitor 2 (Emi2) or c-Mos both exhibit metaphase II arrest defects, but the former fail to establish arrest [32] whereas the latter establish a transient arrest lasting 2–4 h but are unable to maintain this arrest and undergo spontaneous parthenogenetic activation [33, 34].

**Fig 6:**
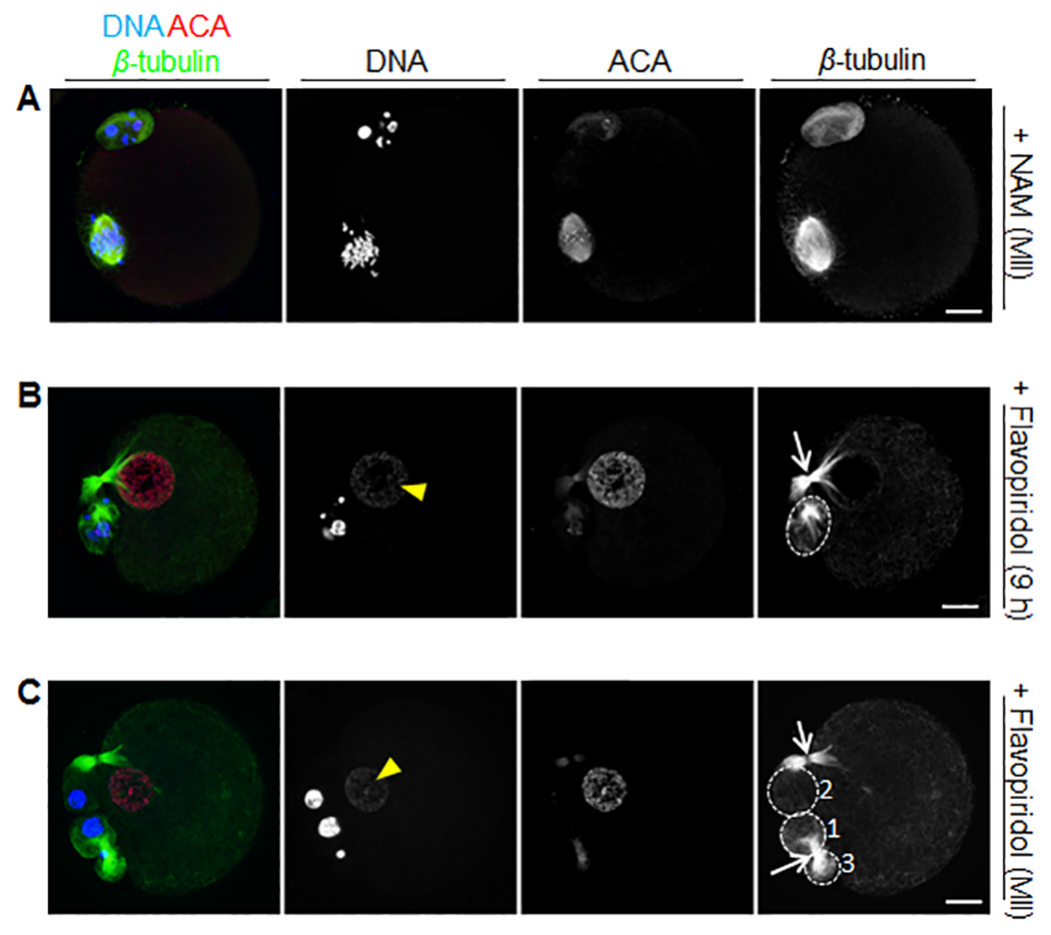
Effect of NAM treatment at metaphase II and of flavopiridol treatment at 9 h post-GVBD and at metaphase II. (A) GV-stage oocytes were matured *in vitro* to metaphase II stage and then incubated in 10 mM NAM for 20 h before being fixed and immunostained. (B, C) GV-stage oocytes were cultured *in vitro* in standard medium either till 9 h post-GVBD (B) or till metaphase II (C) and were then transferred into medium containing 5 μM flavopiridol for 20 h before being fixed and immunostained. Shown are representative confocal images of oocytes from the different treatment groups immunostained for chromosomes (DNA), kinetochores (ACA), and spindle microtubules (β-tubulin). Note that in the NAM-treated oocytes, a well-formed bipolar spindle bearing condensed chromosomes is readily discernible in the oocyte (A). In contrast, following flavopiridol treatment, microtubules form a diffuse network in the oocyte, a persistent central spindle connects oocyte and PB (white arrows) and decondensed chromatin is observed within a nuclear structure (white arrowheads) (B, C). Significantly, in the 9 h flavopiridol group, there is a single PB (B) whereas in the metaphase II flavopiridol group, three PBs are visible (numbered 1–3). The initial PB that was present before flavopiridol was added divided to form PB1 and PB3 following treatment since neither one is connected to the oocyte by a central spindle; interestingly, they remain connected to one another by a persisting central spindle. Following flavopiridol treatment, PB2 was extruded from the oocyte and remains connected via a second central spindle. Only the phenotype in (B) replicates that observed in oocytes treated with NAM from the GV-stage (see Fig 4B). Scale bars = 10 μm.

To differentiate between these two possibilities, we first treated *in vitro* matured metaphase II-arrested oocytes with NAM. We found that 36 of 37 oocytes cultured in 10 mM NAM for >20 h maintained a well formed bipolar spindle with condensed chromosomes (Fig 6A), consistent with previous reports of ovulated mouse eggs treated with 20 mM NAM [9]. Thus, whereas 18–24 h of NAM treatment commencing at the GV-stage induced an interphase-like state (Fig 4B), treatment for similar periods after PBE had occurred did not, suggesting that unlike loss of c-Mos [33, 34], NAM does not compromise maintenance of metaphase II arrest to induce parthenogenesis.

To investigate this further, we next utilized the potent small molecule Cdk1 inhibitor, flavopiridol, which enabled us to rapidly inactivate Cdk1 at specific time-points during meiotic maturation. We used a dose of 5 μM as this effectively inhibits Cdk1 in mouse oocytes [35]. We first treated oocytes at 9 h post-GVBD (9 h flavopiridol, n = 38) and found that chromatin decondensed within a nuclear structure (Fig 6B). Moreover, in 33 of 38 cases there was a clearly identifiable PB that was linked to the oocyte by a persisting central spindle (Fig 6B), identical to the phenotype observed with NAM (see Fig 4B). Next we treated oocytes that had matured *in vitro* to metaphase II with flavopiridol. We found that in these oocytes, flavopiridol also produced decondensed chromatin in a nuclear structure (n = 44; Fig 6C). Significantly however, unlike NAM-treated and 9 h flavopiridol-treated oocytes, at least two PBs were clearly identifiable in 40 of 44 oocytes in this group, and although a central spindle persisted between the oocyte and one of the PBs, there was at least one PB that was not connected to the oocyte by a persisting central spindle (Fig 6C). Thus, forced Cdk1 inactivation during exit from meiosis I, but not at the metaphase II-arrested stage, phenocopied NAM treatment. From this we infer that NAM impairs the establishment rather than the maintenance of metaphase II arrest.

### The Phenotype Observed with NAM Treatment Involves Cdk1 Deregulation during Late Meiosis I Exit and Is Associated with Increased Cyclin B1 Destruction and Inhibitory Cdk1 Phosphorylation

The meiosis I-to-meiosis II transition is critically dependent upon meticulous regulation of Cdk1 activity so that following partial inactivation during meiosis I exit, Cdk1 is rapidly reactivated to catapult oocytes into a second M-phase and avert interphase [5]. The foregoing data show that NAM-treated oocytes fail to enter metaphase II after meiosis I, instead transitioning into interphase, pointing to marked Cdk1 deregulation. Cyclin B1 levels promote Cdk1 activity whilst Cdk1 (Y15) phosphorylation has the opposite effect and is inhibitory [2, 3].

**Fig 7:**
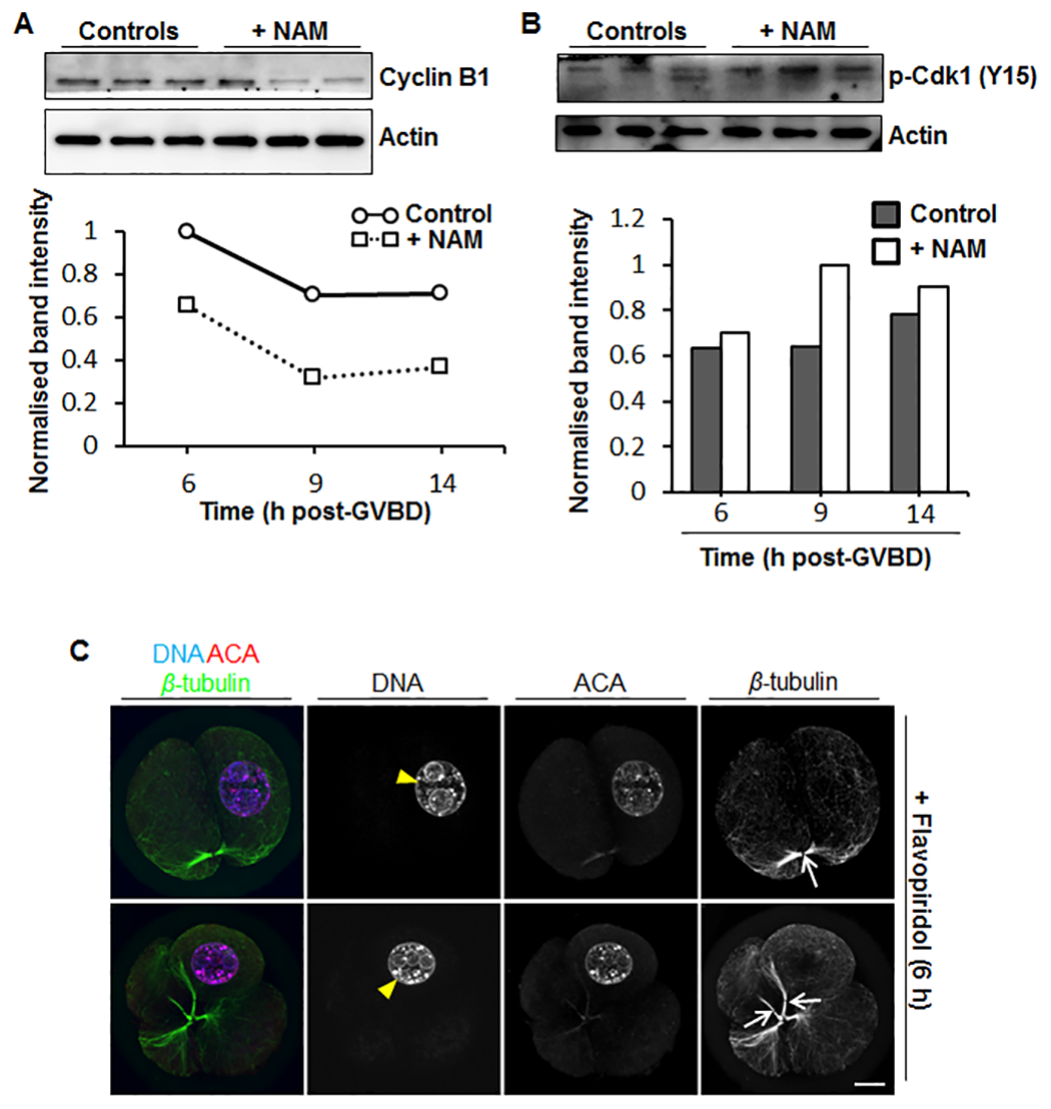
Effect of NAM treatment on cyclin B1 and p-Cdk1 levels and of flavopiridol treatment at 6 h post-GVBD. (A) Western blot of cyclin B1 and actin in control and NAM-treated oocytes at 6 h, 9 h and 14 h post-GVBD (30 oocytes per sample; shown is a representative blot of 3 replicates). Band intensities from Westerns were quantified and normalised against the maximal intensity at 6 h post-GVBD and plotted as a graph. (B) Western blot of p-Cdk1 and actin in control and NAM-treated oocytes at 6 h, 9 h and 14 h post-GVBD (30 oocytes per sample; shown is a representative blot of 3 replicates). Band intensities from Westerns were quantified and normalised against the maximal intensity at 9 h post-GVBD in NAM-treated oocytes. (C) GV-stage oocytes were cultured *in vitro* in standard medium till 6 h post-GVBD and were then transferred into medium containing 5 μM flavopiridol for 20 h before being fixed and immunostained. Shown are representative confocal images of oocytes immunostained for chromosomes (DNA), kinetochores (ACA), and spindle microtubules (β-tubulin). Note that oocytes have either divided into two (top panel) or four (lower panel) similarly sized cells that remain connected via central spindles (white arrows). Note also that chromatin has decondensed and occurs within a nuclear structure (white arrowheads) but that all DNA is retained within one cell. Scale bars = 10 μm.

To examine the effect produced by NAM treatment further, we undertook Western blotting of cyclin B1 and phosphorylated Cdk1 (p-Cdk1) during exit from meiosis I. We studied 3 time-points, 6 h post-GVBD corresponding to the earliest stage of meiosis I exit (Fig 5A), 9 h post-GVBD when exit is well underway (Fig 5A), and 14 h post-GVBD when the majority of oocytes are at metaphase II (Fig 4A). We found that cyclin B1 levels were 40–50% lower in NAM-treated oocytes than in control oocytes at all three time-points (Fig 7A), consistent with the trend towards reduced securin and increased Cdc20 levels we observed previously (Fig 5A and 5B). Although p-Cdk1 levels exhibited a trend towards being higher in NAM-treated oocytes at all three time-points, this difference was most marked (~40% higher) at 9 h post-GVBD (Fig 7B). Thus, following NAM treatment, cumulative changes that would compromise Cdk1 activity to the greatest extent—reduced cyclin B1 and increased p-Cdk1—were most pronounced when meiosis I exit was well underway (9 h post-GVBD).

Late meiosis I exit appeared to be a critical period of Cdk1 deregulation for inducing the morphology observed in NAM-treated oocytes as a very similar phenotype was observed in 9 h flavopiridol oocytes (Fig 6B). In order to explore this further, we investigated the effect of treating oocytes with flavopiridol at 6 h post-GVBD, when exit from meiosis I is at its earliest stage (Fig 5A). Strikingly, flavopiridol treatment at 6 h post-GVBD never resulted in the production of a PB (n = 25) and instead induced symmetrical cleavage into one or more cells (Fig 7C), likely because cytokinesis occurred before the spindle had migrated to the cortex. Furthermore, although chromatin again decondensed within a nuclear structure and a persisting central spindle connected cells, all DNA was nevertheless retained within one of the cells (Fig 7C). This contrasted with NAM-treated and 9 h flavopiridol-treated oocytes in which cytokinesis was asymmetric (producing a PB) and DNA was split between the oocyte and the PB (Figs 4B and 6B).

Taken together therefore, following NAM treatment, excessive Cdk1 inactivation related to reduced cyclin B1 and increased p-Cdk1 levels during a critical period late in meiosis I exit compromised the establishment of metaphase II arrest, inducing an interphase-like state.

## Discussion

Here we examined the effect of NAM on mouse oocytes. NAM is well-known to non-competitively inhibit sirtuins, and has routinely been used to study sirtuin function in somatic cells [6, 7, 19, 21]. We found that treatment with NAM markedly reduced GVBD efficiency such that at least an additional hour was required for NAM-treated oocytes to attain rates comparable with controls. We observed a very similar pattern of inhibition with AGK2, a Sirt2-specific small molecule inhibitor [19, 20], suggesting that the effects produced by NAM on GVBD likely reflect inhibition of at least Sirt2. It is not known whether inhibition of other sirtuins contribute to this phenotype although it appears that Sirt1 inhibition is not involved since GVBD proceeded at completely normal rates in the presence of the Sirt1 inhibitor, Ex527. Data from other papers also indicate that NAM inhibits sirtuins in oocytes and embryos as in somatic cells. Firstly, the potent Sirt1 activator, SRT1720, was found to protect the ovarian follicle reservoir in mice against obesity and this was abolished by NAM [12]. Secondly, treatment with either NAM or one of the Sirt1 inhibitors, sirtinol or BML-210, induced very similar profiles of mouse embryonic developmental arrest following *in vitro* fertilisation [11].

Other important roles are emerging for sirtuins in mammalian oocytes and embryos. For instance, Sirt1 and Sirt3 have been shown to be important for the ability of mouse oocytes and embryos, respectively, to combat oxidative stress [11, 18, 36]. In keeping with this, the Sirt1 agonists, resveratrol or SRT1720, exert protective effects on ovarian follicle numbers and oocyte integrity under detrimental conditions induced by aging, obesity and methylglyoxal-induced oxidative damage [18, 37, 38]. Recent data also highlight important roles for sirtuins in regulating chromosome segregation in oocytes. Thus, using a morpholino-based antisense approach, Sirt2 was shown to be important for spindle assembly, chromosome alignment and the fidelity of chromosome segregation during meiosis I in mouse oocytes [39]. Our findings now identify a role for sirtuins—specifically Sirt2—in modulating GVBD in mouse oocytes. Normal GVBD rates were reported at 3 h following release from chemically induced GV-arrest following depletion of Sirt2 in mouse oocytes [39]. Notably, by 3 h post-release from IBMX we also found that GVBD rates in NAM- and AGK2-treated oocytes were comparable to controls whereas significant differences were observed during the preceding two hours (Fig 1A). By evaluating GVBD at a single 3 h-time-point following Sirt2-knockdown [39], it is possible that important differences at earlier time-points may have been overlooked.

Although GVBD was delayed by treatment with NAM, subsequent events during meiosis I, including spindle assembly and chromosome alignment were unperturbed. Notably, the integrity of these meiotic events accompanied by on-time PBE strongly argue against the possibility that other effects induced by NAM were due to non-specific toxic effects. Furthermore, double the NAM concentration used here induced a beneficial, rather than a detrimental, effect in ovulated mouse eggs [9]. Our findings contrast with Sirt2-specific knockdown in which spindle assembly defects and chromosome misalignment were prominent during meiosis I [39]. Such differences could reflect more severe defects in Sirt2 function induced by morpholino-induced knockdown than achieved using NAM. Unexpectedly, although PBE appeared grossly normal, both in terms of timing and morphology, NAM severely compromised the establishment of the metaphase II arrest-state.

Our data suggest that impairments to GVBD and the establishment of metaphase II arrest in NAM-treated oocytes reflect Cdk1 deregulation. Thus, we observed reduced cyclin B1 accumulation following release from IBMX in NAM-treated oocytes, which would be predicted to dampen Cdk1 activation kinetics and delay GVBD as occurs when increased APC-Cdh1 activity suppresses cyclin B1 levels [40]. Conversely, increased GVBD capacity occurs in the face of higher cyclin B1 levels when APC-Cdh1-mediated destruction is inhibited [15, 41]. It is interesting in this regard that Sirt2, which we find influences GVBD, has recently been shown to modulate the activity of APC-Cdh1 [42]. We found that NAM treatment led to reduced cyclin B1 and increased p-Cdk1 levels during exit from meiosis I, which together, would be predicted to inactivate Cdk1 [2, 3]. Moreover, forced Cdk1 inactivation imposed by flavopiridol during late meiosis I exit induced an interphase-like state with PBE akin to that observed with NAM treatment whereas Cdk1 inactivation earlier during meiosis I or after metaphase II arrest was established did not replicate the phenotype observed with NAM treatment. From this we infer that treatment with NAM led to excessive Cdk1 inactivation during late meiosis I that drove oocytes into interphase and severely compromised establishment of metaphase II arrest.

Metaphase II arrest is due to an activity termed cytostatic factor (CSF), which sustains Cdk1 activity by inhibiting APC-mediated cyclin B1 destruction [4, 5]. Mouse oocytes lacking either Emi2 [32], or Mastl [16] are unable to establish metaphase II arrest, producing a very similar phenotype to that observed here with NAM treatment. This phenotype is distinct from oocytes lacking the proto-oncogene, c-Mos, which arrest transiently at metaphase II but then fail to sustain arrest and undergo parthenogenetic activation [33, 34]. Notably, both Emi2 and Mastl impact APC-Cdc20 activity; the former is an APC-Cdc20 inhibitor whereas the latter is required for APC-Cdc20 activation in late meiosis I [5, 16, 32]. Unlike loss of Mastl, NAM treatment did not compromise APC-Cdc20 activation measured in terms of the timing of securin destruction and SAC inactivation, or Cdc20 levels. Along with the finding that GVBD was unaffected in *Mastl* ^*-/-*^ oocytes [16] therefore, it is unlikely that the defect observed with NAM treatment reflects impaired Mastl function. Impaired Emi2 function cannot fully account for the phenotype observed with NAM treatment either, since Emi2-knockdown oocytes undergo GVBD with normal timing [32]. Although neither deregulated Mastl nor Emi2 appear to account for NAM-induced defects at the meiosis I-to-meiosis II transition, our data nevertheless point to deranged APC-Cdc20 regulation as a contributory factor. Thus, we observed a trend towards increased Cdc20 levels with concomitant reductions in securin and cyclin B1 levels during meiosis I exit, together suggestive of increased APC-Cdc20-mediated proteolysis. Based on available knowledge of sirtuin activity, with Sirt2 shown to be a regulator of APC activity [42], Sirt2 seems a potential candidate for explaining the effects of NAM, especially given the very similar effects that were induced by AGK2 and NAM on GVBD. Although Sirt2-depleted oocytes were most extensively analysed for their meiosis I phenotype, it is interesting that one severe phenotype that was observed following Sirt2 knockdown was a complete absence of a bipolar spindle [39]. In the future, investigation of the effects of NAM treatment on individual sirtuins within oocytes, as well as the impact of sirtuins on the unique pattern of APC activity found in oocytes [22], is warranted to better understand how NAM elicits its effects.
