# Correction: Nicotinamide Impairs Entry into and Exit from Meiosis I in Mouse Oocytes

**DOI:** 10.1371/journal.pone.0130058

**Published:** 2015-06-03

**Authors:** Angelique Riepsamen, Lindsay Wu, Laurin Lau, Dave Listijono, William Ledger, David A Sinclair, Hayden Homer

The sixth author’s name is missing a character. The complete, correct name is: David A Sinclair. The correct citation is: Riepsamen A, Wu L, Lau L, Listijono D, Ledger W, Sinclair DA, et al. (2015) Nicotinamide Impairs Entry into and Exit from Meiosis I in Mouse Oocytes. PLoS ONE 10(5): e0126194. doi:10.1371/journal.pone.0126194
